# Exams disadvantage women in introductory biology

**DOI:** 10.1371/journal.pone.0186419

**Published:** 2017-10-19

**Authors:** Cissy J. Ballen, Shima Salehi, Sehoya Cotner

**Affiliations:** 1 Department of Biology Teaching and Learning, University of Minnesota, Minneapolis, MN, United States of America; 2 Graduate School of Education, Stanford University, Stanford, CA, United States of America; University of Westminster, UNITED KINGDOM

## Abstract

The gender gap in STEM fields has prompted a great deal of discussion, but what factors underlie performance deficits remain poorly understood. We show that female students underperformed on exams compared to their male counterparts across ten large introductory biology course sections in fall 2016 (*N* > 1500 students). Females also reported higher levels of test anxiety and course-relevant science interest. Results from mediation analyses revealed an intriguing pattern: for female students only, and regardless of their academic standing, test anxiety negatively impacted exam performance, while interest in the course-specific science topics increased exam performance. Thus, instructors seeking equitable classrooms can aim to decrease test anxiety and increase student interest in science course content. We provide strategies for mitigating test anxiety and suggestions for alignment of course content with student interest, with the hope of successfully reimagining the STEM pathway as one that is equally accessible to all.

## Introduction

Women who enter college intending to pursue a science, technology, engineering, or mathematics (STEM) discipline leave in greater proportions than their male peers, and remain globally underrepresented in most STEM professions [1–3]. Explanations for the observed female attrition at the college level range from exposure to implicit and explicit bias [4–7], discrimination [5, 8–11], feelings of exclusion in the classroom [12], imposter syndrome [13] and a lack of role models [14, 15]. In addition to lower female retention rates [16], performance disparities between women and men are observed across STEM disciplines, including undergraduate biology [17], physics [18–21], engineering [22], and math [23, 24]. The grade differential may result from female underperformance on exams, a phenomenon that can be explained in full or in part by increased risk perception or test anxiety that prevent some students from retrieving knowledge in an exam environment [25]. Notably, recent studies have verified the role of grade sensitivity in explaining gender imbalances: females students cite low grades and large gateway courses as reasons for declining interest in a discipline compared to male students in equivalent academic standing [26, 27]. If psychological barriers prevent women from performing optimally on exams, it may be time to reconsider exams as a primary method for evaluating student knowledge, particularly if exam performance is not connected to skills necessary for developing STEM professionals.

To explore what factors impact academic performance for women and men in introductory science courses, we addressed four questions: 1) What is the extent of the gender gap in incoming academic preparation among students? 2) What is the extent of the gender gap in exam grades and non-exam grades? 3) Do women and men report different levels of test anxiety and interest in science? 4) Do these two affective factors influence performance outcomes in undergraduate biology courses?

We hypothesized that we would observe men over-performing on high-stakes assessments (e.g., course exams) relative to women, but not on low-stakes summative assessments that contribute to final course grades (non-exam grades; e.g., written assignments, collaborative group work, quizzes). We also hypothesized that an inverse relationship exists between self-reported test anxiety and student performance. Finally, we hypothesized that test anxiety would have a stronger effect on exam performance compared to non-exam assessments.

**Fig 1:**
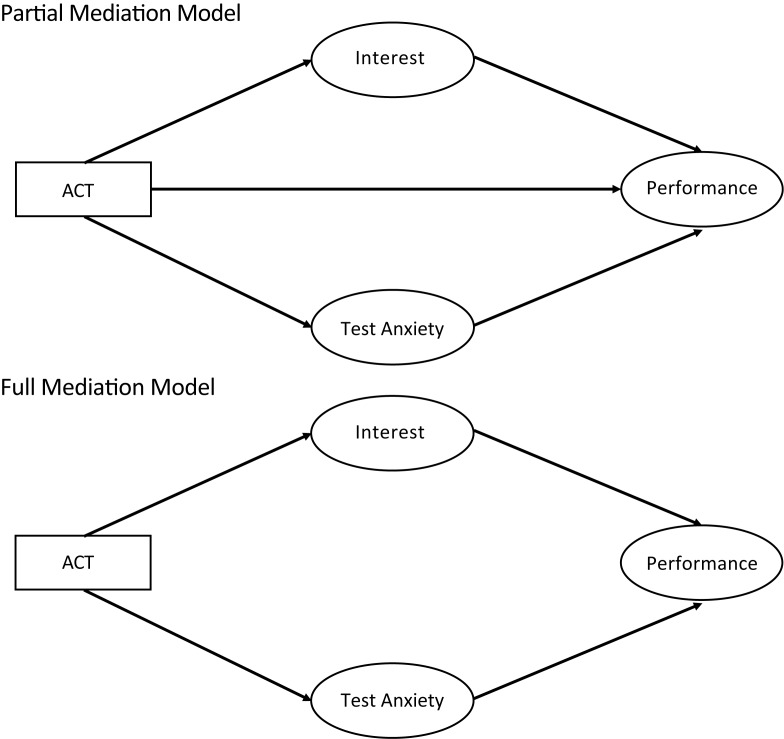
Contrast partial and full mediation models to test mediation effects on student performance. The partial model tests the *partial* mediation effect of science interest or test anxiety on students’ performance. In this model, ACT directly and indirectly via science interest or test anxiety affects students’ performance. The full mediation model tests how incoming preparation (ACT) affects student performance indirectly via science interest or test anxiety of students.

To address our first and second research questions, we examined the relationship between student gender and (1) comprehensive scores on the American College Test (hereafter ACT), which evaluates high school students’ academic preparation for college coursework; (2) combined exam scores and scores on non-exam assessments that contribute to students’ course grades. To address our third and fourth questions, we collected affective measures including interest in science course material and test anxiety (constructs generated from the Motivated Strategies for Learning Questionnaire, or MSLQ; [28]). Using mediation analyses, we examined whether students’ incoming academic preparation (ACT) influences affective measures (test anxiety and interest in course material), which in turn impacts students’ academic performance (Fig 1). We tested whether this mediation effect varies across gender and assessment method.

## Materials and methods

### Biology class preparation and performance

Demographic data were obtained from ten (minimum *N* = 90, maximum *N* = 239) biology courses sections taken by 1562 students (Table 1). We obtained ACT information for *N* = 1205 students (Table 2). We compared (1) combined multiple-choice exam grades; (2) combined non-exam grades e.g., discussion sections, laboratories, online activities, written assignments, low-stakes quizzes, as well as active learning in-class activities. We considered the raw scores of these two components, and then transformed them into z-scores, which represent the distance between the students’ raw score in a given component and the population mean of that component in units of standard deviation (e.g., Z is negative when the raw score is below the mean, positive when above). We calculated z-scores using the formula z-scores = (X - μ) / σ, where X is the score of interest, μ is the class mean score, and σ is the standard deviation.

**Table 1:** Descriptive summary statistics from ten introductory biology courses from fall 2016.

Class section	Instructor	Class N	Women (%)	URM (%)	Average Age (SD)	Average ACT per class (SD)
**A.1**	A	115	61.7	12.2	20.83 (2.47)	25.75 (3.42)
**A.2**	A	115	61.7	8.7	20.70 (2.03)	26.46 (3.55)
**A.3**	B	182	59.9	9.3	20.38 (2.52)	26.62 (2.81)
**B.1**	C	95	44.2	14.7	20.18 (3.15)	26.98 (3.81)
**B.2**	C	90	47.8	16.7	19.68 (1.70)	28.05 (3.17)
**C.1**	D	229	51.5	9.1	20.04 (2.16)	26.89 (3.80)
**D.1**	E,F	153	69.9	12.4	20.29 (2.41)	26.91 (3.55)
**D.3**	E,F	178	58.4	12.4	20.06 (1.86)	26.32 (3.53)
**D.5**	G	239	58.2	14.2	20.18 (2.19)	26.08 (3.55)
**E.1**	H	164	38.8	8.5	20.07 (1.96)	28.69 (3.50)

**Table 2:** The sample of students across ten introductory biology courses who took exams and either had a measure of prior demonstrated academic ability (ACT) that we could obtain from their records or did not have an ACT score.

		Full sample	Sample with a measure of prior demonstrated ability (ACT)	Sample with no measure of prior demonstrated ability (ACT)
*N*		1560	1205	355
Gender	Female	868	687	181
	Male	692	518	174
URM status	URM	180	134	46
	non-URM	1377	1071	306
Median exam percentage (%)		75.05	75.4	71.59
Interquartile range (%)		73.18–76.90	73.55–77.25	69.65–73.49

#### Interest in course content and test anxiety

Before the final exam, we used a validated affective survey to measure aspects of student motivation [28] in three sections of an introductory biology course. Of the 372 students enrolled in these three sections of BIOL 1003, 286 (77%) completed the post-course survey. These data represent 20% of the total students for whom we obtained performance information. Students reported responses using the following scale: 1 = Not at all true of me to 7 = Very true of me. We performed an exploratory factor analysis that resulted in two constructs designed to measure student anxiety during high stakes assessments and interest or perceived usefulness of course content.

For each of these constructs, we had adequate sampling to produce reliable results according to the Kaiser-Meyer-Olkin (KMO) Measure of Sampling Adequacy (KMO > 0.8). We used Bartlett’s test of sphericity to test for the presence of relationships among variables, which were significant for both factors (P < 0.001). Each was highly reliable according to a test for internal consistency (Cronbach’s alpha > 0.7; Table 3). For each construct, we generated a response variable for each student by combining their answers to the loaded questions in that construct using an additive scale. For all Likert scale analyses we treated the dependent variables as continuous [29].

**Table 3:** Data collected from a subset of students in the fall 2016 administration of affective surveys to three different undergraduate biology sections of BIOL 1003 at the University of Minnesota (N = 286).

**Interest in science course content factor (alpha = 0.898)** It is important for me to learn what is being taught in this course. I like what I am learning is this course. I think I will be able to use what I learn in this course in later studies. I think that what I am learning is this course is useful for me to know. I think that what we are learning in this course is interesting. Understanding this subject is important to me.
**Test anxiety factor (alpha = 0.898)** I am so nervous during a test that I cannot remember facts that I have learned. I have an uneasy, upset feeling when I take a test. I worry a great deal about tests. When I take a test I think about how poorly I am doing.

## Statistical analyses

### What is the extent of the gender gap in academic preparation and performance?

We conducted a mixed-effect regression to examine the partial correlation between exam and non-exam grades, while controlling for the effect of gender. We then used a mixed-effects regression to predict the effects of gender on ACT, and to analyze predictors of students’ exam grade and non-exam grade (such as laboratory grade, homework assignments, low stakes quizzes, etc.). The data in this study are hierarchically nested, so we use *multilevel modeling* to account for this non-independence of data in nested-data structures [30, 31] such as lecture sections within course number (e.g., lecture section 10, 20, and 30 within BIOL 1003). We ran the analysis with and without students’ incoming composite ACT scores as a fixed effect, as a proxy for academic preparation. By reporting actual performance rather than model-based estimates that control for pre-scores, we show the actual achievement gaps and that male and female students are earning different grades.

Our research questions in this study mainly focus on the effect of students’ gender and incoming preparation on their performance. To address our research questions, we started with the basic regression model that predicted students’ performance by student gender identity (a factor with two levels; SGender); and their incoming preparation approximated by ACT score. To this basic model, we added the following fixed variables that may contribute to student performance: (1) race/ethnicity/nationality (analyzed as a two-level factor, based on whether a student is from an underrepresented minority [in STEM] group; URM.status); (2) an interaction between student gender identity and URM.status (SGender*URM.status); (3) class size (ClassSize); (4) student academic level (i.e. year in school). To determine the most appropriate model, we then used the Akaike's information criterion (AIC) as a multi-model inference technique [32]. Only students with a complete set of all variables were included in analyses. We ultimately chose the most parsimonious model that best fits the data in accordance to AIC model-selection statistics; this model includes composite ACT score and SGender, model 1 in Table 4.

**Table 4:** Best models for predicting composite *exam* grade using AIC model selection. For non-exam grade, the model that best fit the data also included ACT and SGender, with the next best model including URM.status and ΔAIC = 1.722.

Rank	Model	AIC	ΔAIC
**1**	ACT + SGender	2972.270	0
**2**	ACT + SGender + URM.status	2975.407	3.137
**3**	ACT + SGender + URM.status + SGender*URM	2976.712	4.442
**4**	ACT + SGender + ClassSize	2980.415	8.145
**5**	ACT + SGender + URM.status + ClassSize	2983.484	11.214

#### Do women and men report different levels of test anxiety and interest in science?

Using a subset of students who filled the MSLQ survey (*N* = 286), we performed statistical analyses on affective measures of interest in course science content (‘science interest’) and test anxiety using linear mixed-effects models with the gender and ACT score as the fixed effect and lecture section (BIOL 1003 section 1, 2, and 3) as a random effect. In these analyses, we have normalized the affective measure, so that the regression coefficients are easier to interpret for effect size.

#### Do affective factors influence performance outcomes?

The mediation analyses were conducted using Lavaan R package [33]. In mediation analyses, students’ ACT score affected academic performance through three different paths: one direct path and two indirect paths mediated by science interest and test anxiety (Fig 1). We examined which of these three paths were significant (S1 Appendix). The mediation analysis was conducted separately for exam performance and non-exam mixed assessments performance. To test whether the mediation effect of science interest and test anxiety were different across genders, we used the group analysis option in Lavaan, which allows the coefficients of mediation analysis to be different across gender. For the mediation analysis of both exam grade and non-exam performances, we compared the fit of partial and full mediation models (Fig 1). In the full mediation model, the effect of ACT score on performance is fully mediated by science interest and test anxiety, meaning that ACT score affects performance only indirectly by changing students’ science interest and/or test anxiety. In the partial mediation model, the effect of ACT score on performance is only partially mediated by science interest and/or test anxiety, implying that ACT score both affects performance directly, as well as indirectly by influencing students’ science interest and test anxiety. We found that for both exam performance and non-exam performance, the full mediation model did not fit the data well: The estimated co-variances of this model were significantly different from the actual co-variances in the data (Exam: χ2 (4, *N* = 221) = 72.253, *P* < 0.0001, Non-exam: χ2 (4, *N* = 221) = 16.918, *P* = 0.002). Also none of the other fit indices of the full mediation model fell within the acceptable range [root mean squared (RMSEA): Exam = 0.402, Non-Exam = 0.171 (acceptable range: less than 0.08); comparative fit index (CFI): Exam = 0.204, Non-exam = 0.474 (acceptable range: above 0.95); standardized root mean square residual (SRMR): Exam = 0.158, Non-exam = 0.082 (acceptable range: less than 0.08)][34]. However, the partial mediation model fit the data well for both exam and non-exam performance. The estimated co-variances of the partial mediation model were not significantly different from the actual co-variances in the data [for both exam and non-exam: χ2 (2) = 1.681, p = 0.431]. The other fit indices of the model were also within the acceptable range [for both exam and non-exam: root mean square error (RMSEA) = 0.000 (acceptable range: less than 0.08); comparative fit index (CFI) = 1.000 (acceptable range: above 0.95), standardized root mean square residual (SRMR) = 0.027 (acceptable range: less than 0.08)] (S1 Appendix). This partial model tests the direct effect of students’ ACT on their performance as well as its indirect effect mediated by the affective factors of science interest and/or test anxiety (Fig 1).

## Results

### What is the extent of the gender gap in incoming academic preparation among introductory biology students?

We compared incoming ACT scores of female and male students using a mixed-effect regression model. This analysis revealed a significant difference between genders: ACT scores for women were, on average, 0.28 standard deviation lower than men (*B* = - 0.283, *t (df = 1284)* = 5.178, *P* < 0.0001, *SE* = 0.055).

### What is the extent of the gender gap in exam grades and non-exam grades?

Across course sections, exam and non-exam grades of students were significantly and positively correlated (*B* = 0.387, *t*(1444) = 12.384, *P* < 0.0001, *SE* = 0.031), and this correlation was not significantly different across gender (*B* = 0.068, *t*(1444) = 1.420, *P* = 0.156, *SE* = 0.048). We also found that women underperform on biology exams compared to men (*B* = - 0.146, *t*(1446) = -2.773, *P* = 0.006, *SE* = 0.053), but receive higher non-exam grades than men (*B* = 0.296, *t*(1446) = 5.673, *P* < 0.0001, *SE* = 0.052). These results suggest that women’s exam scores on average was 0.15 standard deviation lower than men, and their non-exam scores were on average 0.3 standard deviation higher than men. When we included incoming ACT score in the model as a fixed effect, the gender gap in exam performance disappeared (*B* = -0.042, *t*(1200) = -0.867, *P* = 0.386, *SE* = 0.049), but women still received significantly higher non-exam grades than men (*b* = 0.297, *t*(1125) = 5.251, *P* < 0.0001, *SE* = 0.056). This means that after controlling for difference in students’ academic preparation, there was no difference between women and men’s exam performance, however women still achieve 0.3 standard deviation higher grades on non-exam assessments. These results suggest that the performance gap on exams in introductory biology can be explained by ACT performance. However, ACT performance does not explain the gender gap on non-exam grades, which show women outperforming men.

### Do women and men report different levels of test anxiety and interest in science?

Across course sections, interest in course-specific science content and test anxiety were not significantly correlated (*B* = 0.124, *t*(272.9) = 1.580, *P* = 0.115, *SE* = 0.079), and this was not significantly different across gender (*B* = -0.088, *t*(273.83) = -0.73, *P* = 0.466, *SE* = 0.121). Furthermore, across course sections and after controlling for students’ ACT score, women reported on average 0.38 standard deviation higher interest in course-specific science content (*B* = 0.375, *t*(222) = 2.774, *P* = 0.006, *SE* = 0.135) and 0.43 standard deviation higher level of test anxiety (*B* = 0.425, *t*(220) = 3.092, *P* = 0.002, *SE* = 0.137) compared to men.

#### Do affective factors influence performance outcomes?

We showed that the observed difference in exam scores between women and men is due to women’s lower incoming academic preparation. To explore the possibility that other variables mediate the effect of incoming preparation on exam performance, we used mediation analyses. Mediating variables transmit effects of an independent variable on a dependent variable, illustrating their structural relationships [35, 36]. We were interested in the mediating effect of affective measures such as science interest and test anxiety as they transmit the effect of incoming preparation on exam performance for women and men (Fig 1).

#### Exam grades

A partial mediation model revealed a correlation between ACT score and academic performance for all students, confirming previous research that demonstrates the same trend [37]. The direct effect of ACT score was stronger on students’ exam grades than non-exam grades. This observation is reasonable because exam performance (and the associated gender gap) mirrors students’ performance on the ACT, which is itself a high-stakes assessment similar to exams. For women, one standard deviation increase in ACT score increased exam grade by 0.55 standard deviation (*P* < 0.0001), and 0.41 standard deviation for men (*P* < 0.0001).

**Fig 2:**
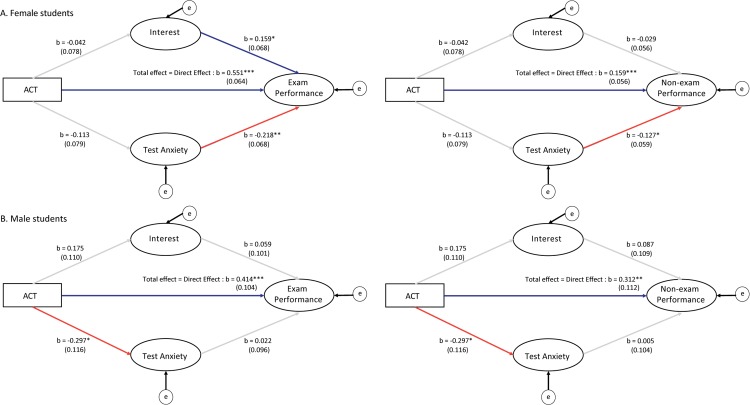
**Partial mediation analyses show differences in the significant effects of incoming preparation (ACT) on exam grade and non-exam grade for (A) female and (B) male students.** Red arrows depict negative effects and blue arrows show positive effects. ACT has direct, positive effects on exam (left) and non-exam (right) grades for all students. For female students, ACT does not influence affective measures such as science interest and test anxiety, but these affective measures influence exam and non-exam grades. For male students, ACT negatively affects test anxiety, but test anxiety does not in turn influence exam and non-exam grades. * p < 0.05, ** p < 0.01, *** p < 0.0001.

We found non-significant indirect effects of ACT on exam grades for female or male students, though for different reasons (Table 5). For women, ACT score did not correlate with interest in science or test anxiety (science interest *P* = 0.59; test anxiety *P* = 0.15). However, science interest and test anxiety both significantly correlated with exam grades; one standard deviation increase in science interest increased women’s exam grade by 0.16 standard deviation (*P* = 0.02); one standard deviation increase in test anxiety decreased women’s exam grade by 0.22 standard deviation (*P* = 0.001; Fig 2). For men, ACT score was correlated with test anxiety (*P* = 0.011), with one standard deviation increase in ACT decreased men’s test anxiety by 0.3 standard deviation. However, decrease in test anxiety did not affect exam performance (*P* = 0.82; Fig 2).

**Table 5:** Summary of partial mediation analysis for exam performance. The numbers in parentheses represent standard errors. Interest, test anxiety, ACT, and exam scores are normalized for ease of interpretation.

	Female	Male	
Models	Coefficient	Coefficient	
	(SE)	(SE)	
Interest ~			
	-0.042	0.175	
ACT	(0.078)	(0.110)	
	*P* = 0.588	*P* = 0.112	
Test Anxiety ~			
	-0.113	-0.297*	
ACT	(0.079)	(0.116)	
	*p* = 0.149	*P* = 0.011	
Exam Performance ~			
	0.159*	0.059	
Interest	(0.068)	(0.101)	
	*P* = 0.019	*P* = 0.563	
	-0.218**	0.022	
Test Anxiety	(0.068)	(0.096)	
	*P* = 0.001	*P* = 0.817	
	0.551***	0.414***	
ACT	(0.064)	(0.104)	
	*P* < 0.0001	*P* < 0.0001	
Structural equation model metrics			
N			221
Df			2
χ^2^			1.681
P (χ^2^)			0.431
RMSEA			0.00
CFI			1.00
SRMR			0.027

* *P* < 0.05

** *P* < 0.01

*** *P* < 0.001

#### Non-exam grades

The partial mediation model shows that the direct effect of ACT scores on students’ non-exam grades was significant for both women and men. For women, one standard deviation increase in ACT score directly increased women’s non-exam grade by 0.16 standard deviation (*P* = 0.004), and 0.31 standard deviation for men (*P* = 0.005).

Similar to exam grades, the indirect effects of ACT score on non-exam grades was not significant for female or male students (Table 6). For women, test anxiety significantly correlated with non-exam grades; one standard deviation increase in test anxiety decreased the non-exam grade by 0.13 standard deviation (*P* < 0.0001). Science interest was not a significant predictor of non-exam grade (*P* = 0.63). For men, the non-exam grade was not correlated with either test anxiety (*P* = 0.96), or with science interest (*P* = 0.43), and thus not correlated with ACT score through either affective measure (Fig 2).

**Table 6:** Summary of partial mediation analysis for non-exam performance. The numbers in parentheses represent standard errors. Interest, test anxiety, ACT, and non-exam scores are normalized.

	Female	Male	
Models	Coefficient	Coefficient	
	(SE)	(SE)	
Interest ~			
	-0.042	0.175	
ACT	(0.078)	(0.110)	
	*P* = 0.588	*P* = 0.112	
Test Anxiety ~			
	-0.113	-0.297*	
ACT	(0.079)	(0.116)	
	*P* = 0.149	*P* = 0.011	
Non-exam Performance ~			
	-0.029	0.087	
Interest	(0.059)	(0.109)	
	*P* = 0.626	*P* = 0.428	
	-0.127*	0.005	
Test Anxiety	(0.059)	(0.104)	
	*P* = 0.031	*P* = 0.964	
ACT	0.159**	0.312**	
	(0.056)	(0.112)	
	*P* = 0.004	*P* = 0.005	
Structural equation model metrics			
N			221
Df			2
χ^2^			1.681
P (χ^2^)			0.431
RMSEA			0.00
CFI			1.00
SRMR			0.027

* *P* < 0.05

** *P* < 0.01

## Discussion

Using student data from ten introductory biology course sections in fall 2016, we demonstrate that women underperformed on ACT and exams as compared to their male counterparts, but outperformed men on combined non-exam methods of assessment. Mediation analyses revealed two further findings: for men, ACT score was not correlated with science interest, and science interest did not influence exam grade. For women, however, ACT score was not correlated with science interest, while science interest significantly influenced exam performance. Second, for men, though ACT score was correlated with test anxiety, test anxiety did not influence exam grade. For women, ACT scores did not correlate with test anxiety, but test anxiety significantly influenced exam grade (Fig 2).

Our results suggest that instructor efforts to design curricula that promote students’ interest can positively impact exam performance, particularly for women. Furthermore, these efforts will benefit female students regardless of their incoming preparation. Previous research shows that encouraging students to connect course material to their lives increases interest and performance in science courses early in high school [38]. Gender differences in attitudes towards and interest in science [39] means that making course content personally relevant for both women and men might be a challenging task. However, these efforts are particularly important in male-dominated academic areas (e.g., math, physics, or engineering) where women are underrepresented and more likely to consider changing their major [40].

Our results also show one measure of academic preparation, ACT score, accurately predicts test anxiety for men in college. However, for women, this prior demonstrated competency do not predict test anxiety. In addition, for women only, increasing test anxiety has a significant and sizeable negative impact on exam performance: one standard deviation increase in test anxiety decreases the exam grade by 0.28 standard deviation. This effect is almost half the size of that for incoming preparation: one standard deviation increase in ACT score increases the exam grade by 0.55 standard deviation. Our findings underscore the likelihood that performance during high-pressure testing may not reflect actual content knowledge for some underrepresented groups [41, 42]. For women, test anxiety may stem from social psychological barriers such as stereotype threat [40, 43, 44], whereby in high-stakes testing situations (i.e. high-value course exams) females experience a self-evaluative apprehension of conforming to the perceived stereotype of female inferiority in STEM subjects.

If test anxiety, coupled with stereotype threat, is culpable in the underperformance of women on high-stakes exams, efforts to minimize threat during exams should reduce the gender differences we, and others, have documented in STEM disciplines. This hypothesis and associated predictions are testable and, if our predictions are correct, the actionable items are simple to implement. Instructors could minimize the impact of high-stakes tests by offering a diversity of assessment types in their courses. For example, active learning is defined in part by its use of formative and summative assessment methods, and evidence for performance gains in active-learning environments is compelling and broad [45–48]. Techniques vary, but active learning can include group work, case studies, modeling exercises, and a diversity of in-class assessment techniques (e.g., classroom response systems, Immediate Feedback Assessment Technique forms, worksheets, and one-minute papers). Incentivizing students to participate through mixed methods of assessment rewards consistent, ongoing preparation rather than performance on a few high-stakes examinations. We hypothesize that mixed assessment methods in active learning classrooms serve as relatively nonthreatening opportunities for females and others to demonstrate knowledge under minimized susceptibility to test anxiety, thus increasing females’ overall performance. In this study, the negative effect of test anxiety on females’ performance was twice as high for exams as it was for non-exam grades (Table 6). Thus, incorporation of mixed assessment methods may be particularly beneficial in male-stereotyped STEM fields where women are a minority in the classroom and suffer the largest susceptibility to stereotype threat in test environments [43, 49].

One limitation of this study that may influence the interpretation of our findings is the possibility that our survey instruments functioned differently for the different groups of students we sampled. While we examined exclusively non-majors’ introductory biology lecture courses, we still observed course-specific differences in classroom demographic composition and student preparation (Table 1). As future research broadens in scope to examine student populations across STEM fields, it will become increasingly important to compare different groups of students’ responses to survey instruments. We may also expect that the performance impacts of test anxiety and course interest will change based on discipline.

Although our emphasis is on differential performance as a function of gender, we anticipate similar phenomena may characterize the experiences of underrepresented minority students, first-generation college students, and any student more susceptible to test anxiety in high-stakes exam environments. The traditional learning environment is not designed for a diverse student body and does not recognize student variation on many dimensions of learning. Evaluating students based primarily on high-stakes exams does not nurture individual potential, and its use to assess our increasingly diverse talent pool will perpetuate existing disparities. Although many uncertainties remain, recent work is beginning to fill in some of the major gaps in our understanding of the effects of tests on underrepresented groups in STEM (e.g., see [50]). We now have plausible hypotheses about the forces responsible, not only with respect to underlying mechanisms [51], but also ways to develop curricula that promote performance of at-risk students [18, 45–48]. The challenging task of robustly testing our hypotheses is still in its infancy, but recent progress is encouraging. Techniques to experimentally manipulate critical parameters (such as writing exercises or teaching with multiple low-stakes assessments) are feasible and should provide increasingly powerful methods to clarify the consequences of different types of assessments for all STEM students. Ultimately, fundamental changes in how we assess mastery in STEM courses may be critical for making the STEM disciplines accessible to all.

## Supporting information

S1 Appendix Mediation analyses. (DOCX)
