# Supplementary material for: Exams disadvantage women in introductory biology
Source: PLoS One. 2017 Oct 19;12(10):e0186419. doi: 10.1371/journal.pone.0186419 (PMC5648180; doi:10.1371/journal.pone.0186419)
Supplement: S1 Appendix — (DOCX) [file pone.0186419.s001.docx]

**Supplementary Material**

**Exams disadvantage women in introductory biology**

Cissy Ballen^1,2^, Shima Salehi^3^, and Sehoya Cotner^1^

*^1^Department of Biology Teaching and Learning, University of Minnesota, Minneapolis, MN, USA*

*^2^E-mail: balle027@umn.edu*

*^3^Graduate School of Education, Stanford University, Stanford, CA 94305*

**S1 Appendix. Mediation analyses.** For both exam and non-exam grades, the full mediation model (Fig 1) did not show a good fit for the data (Table S1) for either gender. By including the direct effect of the ACT on performance, we tested the fit of the partial mediation model (Fig 1). When direct effect of ACT was added to the model, the fit of the mediation model improved drastically for both exam and non-exam grades, and all the fit indices fell within the acceptable range (Table S1). Therefore, in order to most accurately predict students’ performance, the direct effect of ACT score should be included in the mediation model. In the partial mediation model, beside the direct effect of ACT score on performance, we test whether a significant indirect effect of students’ incoming preparation influences their performance. We do this by examining whether students’ ACT changes their interest in course content (interest in science as used earlier) or test anxiety, and whether that influences academic performance (exam and non-exam grades) for females and males.

**S1 Table. Full and partial model fit for mediation analyses of female and male students***.* The full mediation model, whereby ACT score predicts performance indirectly through affective measures, did not show a good fit for the data. The partial mediation model includes the direct effect of the ACT on performance, and for this model all the fit indices fell within the acceptable range.

| **Full mediation model for exam grade**: χ2 (4, *N* = 221) = 72.253, *P* < 0.0001, root mean square error (RMSEA) = 0.402 (acceptable range: 0-0.08), comparative fit index (CFI) = 0.204 (acceptable range: above 0.95), standardized root mean square residual (SRMR) = 0.158 (acceptable range: 0-0.08).  **Full mediation model for non-exam grade**: χ2 (4, *N* = 221) = 16.918, *P* = 0.002, root mean square error (RMSEA) = 0.171 (acceptable range: 0-0.08), comparative fit index (CFI) = 0.474(acceptable range: above 0.95), standardized root mean square residual (SRMR) = 0.082 (acceptable range: 0.08). |
| --- |
|  |
| **Partial mediation model for exam grade**: χ2 (2, *N* = 221) = 1.681, *P* = 0.431, root mean square error (RMSEA) = 0.00 (acceptable range: 0-0.07), comparative fit index (CFI) = 1 (acceptable range: above 0.95), standardized root mean square residual (SRMR) = 0.027 (acceptable range: 0-0.1).  **Partial mediation model non-exam grade**: χ2 (2, *N* = 221) = 1.681, *P =* 0.431, root mean square error (RMSEA) = 0.00 (acceptable range: 0-0.08), comparative fit index (CFI) = 1.00 (acceptable range: above 0.95), standardized root mean square residual (SRMR) = 0.027 (acceptable range: 0-0.08). The better fit of partial mediation model confirms that incoming preparation of students has a direct effect on their performance. |
